# Epidemiological and entomological studies of malaria transmission in Tibati, Adamawa region of Cameroon 6 years following the introduction of long-lasting insecticide nets

**DOI:** 10.1186/s13071-021-04745-y

**Published:** 2021-05-08

**Authors:** Lionel Brice Feufack-Donfack, Elangwe Milo Sarah-Matio, Luc Marcel Abate, Aline Gaelle Bouopda Tuedom, Albert Ngano Bayibéki, Christelle Maffo Ngou, Jean-Claude Toto, Maurice Marcel Sandeu, Carole Else Eboumbou Moukoko, Lawrence Ayong, Parfait Awono-Ambene, Isabelle Morlais, Sandrine Eveline Nsango

**Affiliations:** 1 Service de Paludisme du Centre Pasteur Cameroun, BP 1274, Yaounde, Cameroon; 2 CNRS UPR 9022, Inserm U 963, Université de Strasbourg, 2, allée Konrad Roentgen, 67084 Strasbourg Cedex, France; 3 UMR MIVEGEC, IRD, CNRS, Université de Montpellier, Institut de Recherche pour le Développement, 911 avenue Agropolis, 34394 Montpellier, France; 4 Université Catholique d’Afrique Centrale, Yaoundé-Campus Messa, BP 1110, Yaounde, Cameroon; 5 Laboratoire de Recherche sur le Paludisme, Organisation de Coordination pour la lutte contre les Endémies en Afrique Centrale, BP 288, Yaounde, Cameroon; 6 Department of Medical Entomology, Centre for Research in Infectious Diseases, Yaounde, 13591 Cameroon; 7 Department of Microbiology and Infectious Diseases, School of Veterinary Medicine and Sciences, University of Ngaoundere, PO Box 454, Ngaoundere, Cameroon; 8 Faculté de Médecine et des Sciences Pharmaceutiques de l’Université de Douala (FMSP–UD), BP 2701 Douala, Cameroon

**Keywords:** Malaria, *Plasmodium vivax*, *Anopheles coluzzii*, Epidemiology, Entomology, Cameroon

## Abstract

**Background:**

Malaria remains a serious public health problem in Cameroon. Implementation of control interventions requires prior knowledge of the local epidemiological situation. Here we report the results of epidemiological and entomological surveys carried out in Tibati, Adamawa Region, Cameroon, an area where malaria transmission is seasonal, 6 years after the introduction of long-lasting insecticidal bed nets.

**Methods:**

Cross-sectional studies were carried out in July 2015 and 2017 in Tibati. Thick blood smears and dried blood spots were collected from asymptomatic and symptomatic individuals in the community and at health centers, respectively, and used for the molecular diagnosis of *Plasmodium* species. Adult mosquitoes were collected by indoor residual spraying and identified morphologically and molecularly. The infection status of *Plasmodium* spp. was determined by quantitative PCR, and positivity of PCR-positive samples was confirmed by Sanger sequencing.

**Results:**

Overall malaria prevalence in our study population was 55.0% (752/1367) and *Plasmodium falciparum* was the most prevalent parasite species (94.3%), followed by *P. malariae* (17.7%) and *P. ovale* (0.8%); 92 (12.7%) infections were mixed infections. Infection parameters varied according to clinical status (symptomatic/asymptomatic) and age of the sampled population and the collection sites. Infection prevalence was higher in asymptomatic carriers (60.8%), but asexual and sexual parasite densities were lower. Prevalence and intensity of infection decreased with age in both the symptomatic and asymptomatic groups. Heterogeneity in infections was observed at the neighborhood level, revealing hotspots of transmission. Among the 592 *Anopheles* mosquitoes collected, 212 (35.8%) were *An. gambiae*, 172 (29.1%) were *An. coluzzii* and 208 (35.1%) were *An. funestus* (*s.s.*). A total of 26 (4.39%) mosquito specimens were infected by *Plasmodium* sp. and the three *Anopheles* mosquitoes transmitted *Plasmodium* at equal efficiency. Surprisingly, we found an *An. coluzzii* specimen infected by *Plasmodium vivax*, which confirms circulation of this species in Cameroon. The positivity of all 26 PCR-positive *Plasmodium*-infected mosquitoes was successively confirmed by sequencing analysis.

**Conclusion:**

Our study presents the baseline malaria parasite burden in Tibati, Adamawa Region, Cameroon. Our results highlight the high malaria endemicity in the area, and hotspots of disease transmission are identified. Parasitological indices suggest low bednet usage and that implementation of control interventions in the area is needed to reduce malaria burden. We also report for the first time a mosquito vector with naturally acquired *P. vivax* infection in Cameroon.

**Graphical abstract:**

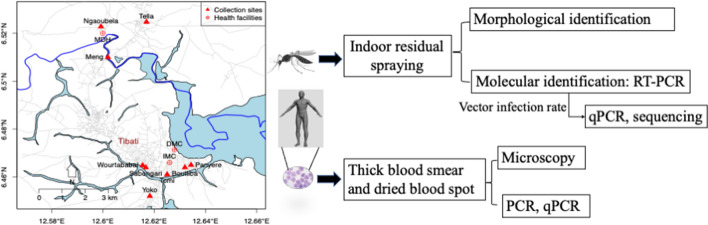

## Background

Malaria remains a deadly scourge in many parts of the world, with 409,000 deaths from this disease reported globally in 2019. Over the past 10 years, a declining trend has been observed in the global incidence of cases and deaths due to malaria, 57 and 10%, respectively [1]. Disease control efforts rely at least in part on vector control measures, in particular the use of long-lasting insecticidal-treated bed nets (LLINs) and indoor residual spraying (IRS). Many countries are now adopting elimination strategies as part of their malaria operational plans, even if malaria burden has remained unchanged in some countries in sub-Saharan Africa. Unfortunately, the African region continues to account for over 90% of all malaria deaths [1].

In Cameroon, malaria remains a serious public health problem. Transmission is heterogeneous across the country, ranging from perennial transmission in the southern forested regions to seasonal and unstable transmission in the northern Sudano-savannah and Sahelian regions. Of the 52 formally recognized *Anopheles* species present in Cameroon, only 16 are able to transmit the disease to humans [2–4], and the most common and efficient vector species are *Anopheles gambiae*, *An. coluzzii*,* An. arabiensis*, *An. funestus*, *An. nili* and *An. moucheti* [5, 6]. Over the past 9 years there has been significant progress in terms of malaria control and prevention. Indeed, from 2010 to 2019, the morbidity and mortality due to malaria decreased from 36 to 28% and from 31 to 18.3%, respectively; however, the situation is not yet under control as the whole country is still at risk of disease transmission [7, 8]. These observed reductions are due to the efforts of the Government of Cameroon and international partners, with a heavy reliance on mass distribution campaigns of LLINs and the use of sulfadoxine/pyrimethamine either alone for intermittent preventive treatment in pregnant women or in combination with amodiaquine for seasonal malaria chemoprevention in children aged < 5 years. The introduction of artemisinin-based combination therapies (ACTs) for the treatment of uncomplicated malaria and of free healthcare management of severe malaria cases in children aged < 5 years have also contributed to malaria reduction [8].

Nevertheless, the effectiveness of control measures is threatened by the rapid expansion of insecticide resistance in vector populations [9–13], changes in vector host-seeking, biting and resting behaviors [14, 15], the emergence and rapid spread of drug-resistant parasite strains [16–20] and the diversity of the vectorial system [2] and parasite species [21, 22]. In Cameroon, four *Plasmodium* species are known at the present time to cause malaria, among which *P. falciparum* is considered to be the most prevalent and virulent parasite, responsible for 82–100% of all malaria cases depending on the locality [23–26]. Other human-infecting *Plasmodium* are also present in Cameroon, including *P. malariae*, *P. ovale* and *P. vivax* [27–30]*.* The latter is the most geographically widespread malaria parasite that causes disease in humans outside of Africa and is especially prevalent in Southeast Asia and the Americas [31, 32]. Individuals in western, eastern and central Africa have long been considered refractory to infection by this parasite because of the high prevalence of the Duffy-negative phenotype [33, 34]. This phenotype is caused by a single nucleotide polymorphism (SNP) in the erythroid-specific promoter region of the DARC (Duffy antigen receptor for chemokines) gene [35], and individuals who are homozygous for the allele carrying this SNP lack the Duffy protein on erythrocytes. However, the dogma of “*Pv* absence in Africa” [36] began to fail a decade ago when evidence began accumulating for *P*. *vivax* infections in Duffy-negative individuals across Africa, and in the intervening time different studies have reported *P. vivax* infections in Mauritania [37, 38], Ethiopia [39, 40], Equatorial Guinea [41], Mali [42], Democratic Republic of Congo [43], Benin [44], Nigeria [45] and Cameroon [27–30]. In Cameroon, the association of *P. vivax* with human infections in both Duffy-positive and -negative individuals has been reported, albeit at low frequencies. However, no study has yet looked at which local malaria vector species is involved in the transmission of *P. vivax.*

In this study, we aimed to characterize malaria parasites and vectors circulating in an area of seasonal transmission in the Adamawa region. We focused on *P. vivax* detection as this species was recently reported in other parts of the country, and we conducted entomological surveys to identify its putative vector, which has not been investigated in earlier studies. Our main goal was to examine the potential risk of transmission of *P. vivax* and to determine which local malaria vector species could be implicated in its transmission in this region of Cameroon.

## Methods

### Study area

The study was conducted in the town of Tibati (6°27′57″N; 12°37′30″E), which is the capital of Djerem department in the Adamawa region of Cameroon. The population of Tibati is estimated to be 108,502 persons. The town is characterized by a predominantly wet tropical climate with two seasons, a dry season extending from November to February and a rainy season from March to October during which the intensity of transmission is high. The annual rainfall ranges from 1192 to 2023 mm, and the average annual temperature is around 23.6 °C. There is a dense hydrographic network of several rivers, such as the Djerem, Meng and Tomi rivers, in the area around Tibati [46].

**Fig. 1:**
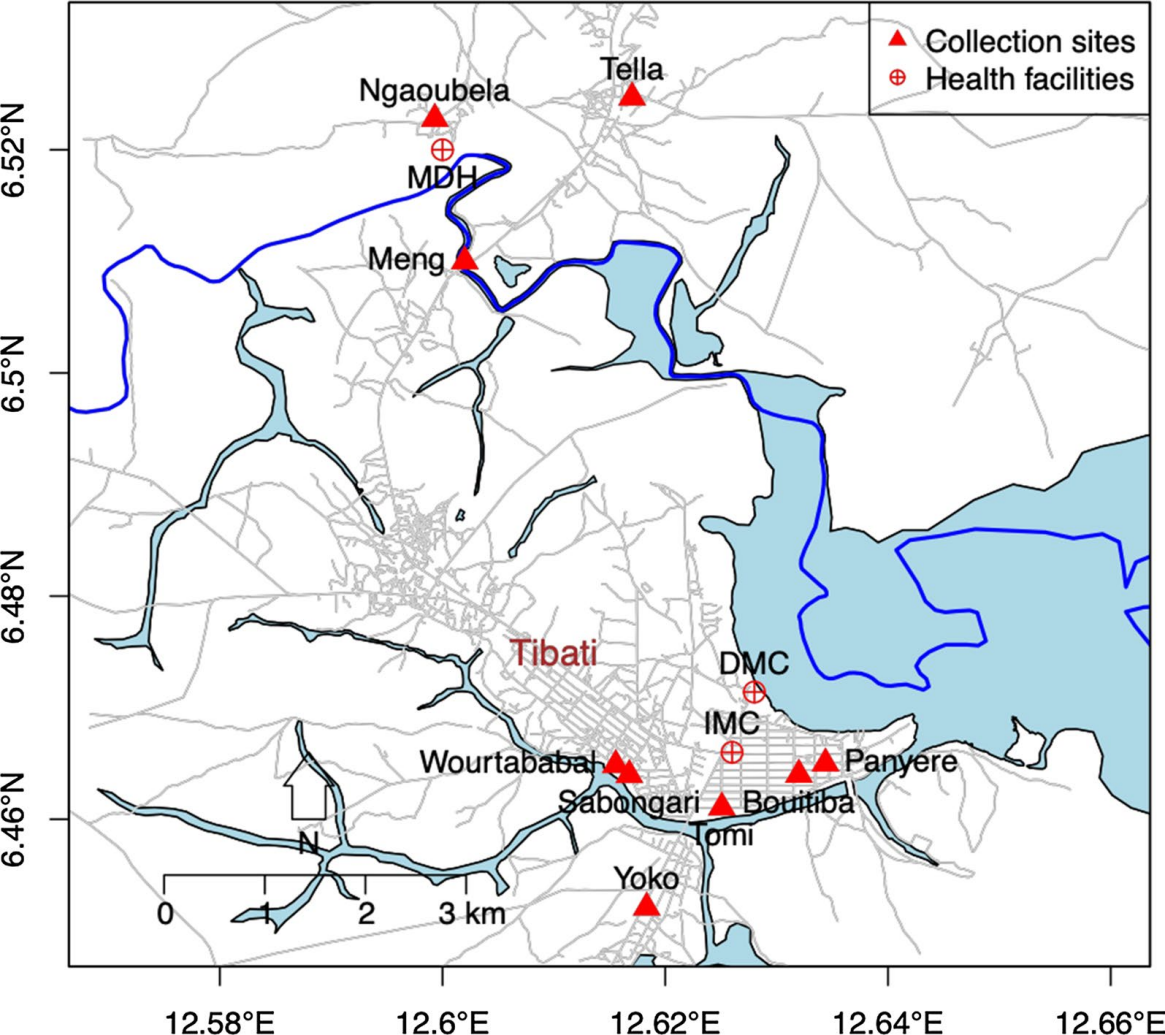
Map of Tibati showing collection sites. Blood samples were collected from symptomatic malaria patients in three health facilities: the Missionary hospital of Ngaoubela (*MDH*), District Medical Center (*DMC*) and the Integrated Medical Center (*IMC*). Blood samples were collected from asymptomatic persons in the community in eight neighborhoods of Tibati: Malarba, Meng, Ngaoubela, Sabongari, Tella, Wourtababa, Tomi and Yoko, which were the same neighborhoods that mosquito collections were performed

The parasitological and entomological surveys were carried out from June to July 2015 and during the same period in 2017, to coincide with the rainy season during which transmission is highest. Blood samples were collected from clinical patients (symptomatic) with fever or a history of fever in 2015 and from asymptomatic individuals in the community in 2017. Patients with clinical malaria were recruited among patients in three randomly selected health facilities: the Missionary Hospital of Ngaoubela, the District Medical Center and the Integrated Medical Center located at around 7 km, 600 m and 100 m from the city center, respectively. The minimal estimated sample size of 346 participants was determined by the formula *n* = *T*^2^ × *P*(1 − *P*)/*M*^2^, where* n* = sample size; *T* =  confidence level at 95% (with value at 1.96); *P* = malaria prevalence in the Adamawa region (34.2%) according to the Programme National de Lutte contre le Paludisme 2019 [8]); and *M* = margin of error at 5% (0.05). Blood from asymptomatic carriers was collected from individuals living in randomly selected houses in the community, from eight neighborhoods within the Tibati city perimeter (Malarba, Meng, Ngaoubela, Sabongari, Tella, Wourtababa, Tomi and Yoko) (Fig. 1). Mosquito collection was performed by spraying resting female mosquitoes in randomly selected human dwellings and in houses where blood from asymptomatic carriers was collected, with IRS with pyrethrum in 2015 and 2017, respectively.

### Blood and mosquito sampling

Blood was collected from volunteers of all ages independently of gender and ethnicity. Adults (≥ 18 years old) or legal guardians were asked to provide written informed consent for underaged volunteers (< 18 years old). Blood was obtained by fingerprick in volumes of 5 and 100 µl for thick smears films and dried blood spots on Whatman™ grade 1 and FTA paper (GE healthcare UK Ltd., Amersham, UK), respectively. Thick blood smears were stained with 10% Giemsa for 20 min [19] and examined under a light microscope (Leica model DM750; Leica Microsystems GmbH, Wetzlar, Germany) at 100× magnification, oil immersion, for the detection of asexual and sexual stages of the malaria parasite. Parasitemia was estimated by counting the number of parasites against 500 white blood cells (WBCs), assuming the standard number of 8000 WBC/µl of blood [47]. Blood spots were dried, stored at room temperature in a desiccant container and brought to the Malaria Research Unit for further analysis. *Plasmodium*-positive patients were treated with an ACT according to the recommendations of the Ministry of Health of Cameroon (National Malaria Control Program [NMCP]).

Mosquito collections were carried out in the morning from 6:00 to 10:00 a.m., and written informed consent was obtained from household owners prior to the start of collection. A white sheet was used to cover the floor and all flat surfaces, and the pyrethrum residual (composition: permethrin, 0.25%; piperonyl butoxide, 0.34%; tethramethrin, 0.20%; d-phenothrin, 0.01%) was sprayed into all the corners of the house. After 15 min, mosquitoes that had fallen onto the sheet were collected, counted and identified at the genus or species level. *Anopheles* mosquitoes were identified using morphological identification keys [48, 49]. All female *Anopheles* specimens were dissected individually, and the carcasses and head-thoraces were stored separately in tubes containing a desiccant, archived and kept at − 20 °C for further molecular analysis.

### Blood and mosquito DNA extraction

DNA was isolated from blood spots collected in 2015 using the protocol provided by the manufacturer (Schleicher-Schuell BioScience GmbH, Dassel, Germany) [50]. Briefly, three 5-mm discs of a dried blood spot were cut out using a sterile paper punch and transferred to a 1.5-ml sample tube filled with 500 µl of sterile water. The tubes were vortexed three times for at least 5 s each time and centrifuged at 2000 rpm for few seconds. Using sterile forceps, the paper discs were transferred into a 0.5-ml tube containing 120 µl sterile water and incubated at 95 °C in a heat block for 15 min (Gene Amp®; Applied Biosystems, Foster City, CA, USA). After incubation, the tubes were centrifuged for few seconds, following which the spots were removed, and the extracted DNA was stored at − 20 °C for molecular diagnosis.

To optimize the DNA extraction and allow for a better separation of DNA from some nucleases, we used the Qiagen DNeasy® Blood and Tissue Kit (Hilden, Germany) to isolate DNA from the blood samples collected in 2017, in accordance with the manufacturer’s instructions, and resuspended the isolated DNA in 60 µl elution buffer.

Genomic DNA from individual mosquito carcasses was isolated using the 2% cetyltrimethyl ammonium bromide (CTAB) method, following the protocol of Collins et al. [51]. DNA from the head-thoraces was isolated using the Qiagen DNeasy® Blood and Tissue Kit according to the manufacturer’s instructions, and resuspended in 50 µl elution buffer.

### Molecular identification of *Plasmodium* and *Anopheles* species

The *Plasmodium* infection status of blood collected in 2015 was determined by a multiplex PCR [52], which targets the 18S small subunit ribosomal RNA. Reaction mixtures were prepared with 5 μl of eluted DNA and the Taq Hot Start Master mix (Qiagen), following the manufacturer’s instructions, and samples were amplified as previously described [52]. The amplified products were visualized by electrophoresis in a 2% agarose gel and stained with SYBR dye (Green Nucleic Acid stain; Biotium, Hayward, CA, USA). The expected amplicon size was 276 bp for *P. falciparum*, 300 bp for *P. vivax*, 375 bp for *P. ovale* and 412 bp for *P. malariae*.

Genomic DNA from mosquito carcasses was used to identify members of the *Anopheles gambiae* complex by PCR–restriction fragment length polymorphism (RFLP) [53] and members of *Anopheles funestus* group were identified according to the protocol of Koekemoer et al. [54]. All PCR products were analyzed in a 2% agarose gel.

A simple quantitative PCR (qPCR) was performed to identify *Plasmodium*-infected mosquitoes, as previously described [55]. Selected genes were amplified in order to discriminate between *Plasmodium* species, including the aquaglyceroporin gene (AQP, AJ413249.1) for *P. falciparum*, the enoyl-acyl carrier protein reductase gene (ECPR, AY423071.1) specific for *P. vivax*, the P25 ookinete surface protein gene (Pos25, AB074976.1) for *P. ovale* and the circumsporozoite gene (CS, S69014) targeting *P. malariae* [56]. Reaction mixtures were performed with 1 μl of template DNA in a final volume of 10 μl with EvaGreen® (5× HOT Pol EvaGreen® RT PCR Mix Plus; Euromodex, Souffelweyersheim, France) and amplified in a 7300 Real-time PCR system (Applied Biosystems). A dissociation curve was used to estimate the specific melting temperature for each reaction.

For the samples from 2017,* An. gambiae* complex and the members of *An. funestus* group were identified as described above. Detection of *Plasmodium* in blood spots and mosquito head-thoraces for these samples was achieved by multiplex qPCR in the Lightcycler 96 real-time PCR system, according to the protocol published by Mangold et al. [57], with slight modifications. The final volume of the reaction mixture was 10 μl, containing 2 μl of template DNA. The PCR conditions consisted of an initial preincubation step at 95 °C for 10 min; followed a three-step amplification of 95 °C/10 s (ramp 4.4 °C/s), 50 °C/5 s (2.2 °C/s) and 72 °C/20 s (4.4 °C/s), for 45 cycles. Amplification was directly followed by a melting program of 95 °C/120 s (2.2 °C/s), 68 °C/120 s (2.2 °C/s) and 90 °C/1 s (ramp 0.2 °C/s with 15 readings/°C), and a stepwise temperature increase of 0.03 °C/s until 95 °C**.** The 18S rRNA gene of the *P. falciparum* 3D7 clone was used as positive control for *Plasmodium* species differentiation.

### Sequencing

The qPCR products of *Plasmodium-*positive mosquitoes were sequenced using the Big Dye Terminator v3.1 Sequencing Kit (Applied Biosystems) and run on an Applied Biosystems 3130xl Sequencer at the GenSeq technical facility of the Institut des Sciences de l’Evolution de Montpellier. Sequences were verified using SeqScape software (Applied Biosystems).

### Statistical analysis

Data were stored in Microsoft Office Excel files (Microsoft Corp., Redmond, WA, USA) and transferred into GraphPad Prism 7 (GraphPad Software Inc., San Diego, CA, USA) for statistical analyses. The Mann–Whitney test was used to compare mean parasite densities according to clinical status, for both trophozoites and gametocytes. The Chi-square test was used to compare the prevalence of *Plasmodium* infections among population age groups and mosquito species. The non-parametric Kruskal–Wallis test was used to assess differences in parasite densities between age groups. The significance threshold was set at alpha = 0.05. The relative risk (RR) was computed to estimate the protection associated with LLIN use. R software version 3.5.3 with maptools and ggplot2 packages was used to generate the map and figures, respectively (R Foundation for Statistical Computing, Vienna, Austria), while melting temperatures of qPCR-positive controls were retrieved by using functional bases (without package).

## Result

### Characteristics and prevalence of malaria infections in the studied population

A total of 1367 participants were enrolled in this study, 418 through the health centers (symptomatics) and 949 at the community level (asymptomatics). The main characteristics of the participants are detailed in Table 1.

**Table 1:** Characteristics of the study population and infection parameters

Parameters	2015	2017
	Health centers (symptomatics)	Community (asymptomatics)
Sample size	418	949
Median age, years (IQR)	15 (3–30)	12 (6–23)
Infections per age group,* n* (%):		
< 5 years	83 (58.5)	110 (62.1)
5–15 years	40 (58.8)	310 (71.4)
> 15 years	52 (25)	157 (46.4)
Total number persons infected	175 (41.8)	577 (60.8)
*Plasmodium* infections,* n* (%)		
* P. falciparum*	173 (98.8)	441 (76.4)
* P. malariae*	1 (0.6)	39 (6.8)
* P. ovale*	1 (0.6)	1 (0.2)
Co-infections	0	96 (16.6)
Mean trophozoite density (/µl) (SD)	34,164 (63,541)	5964 (18,429)
Mean gametocyte density (/µl) (SD)	6199 (18,956)	38.75 (97.2)

*IQR* interquartile, *SD* standard deviation

**Fig. 2:**
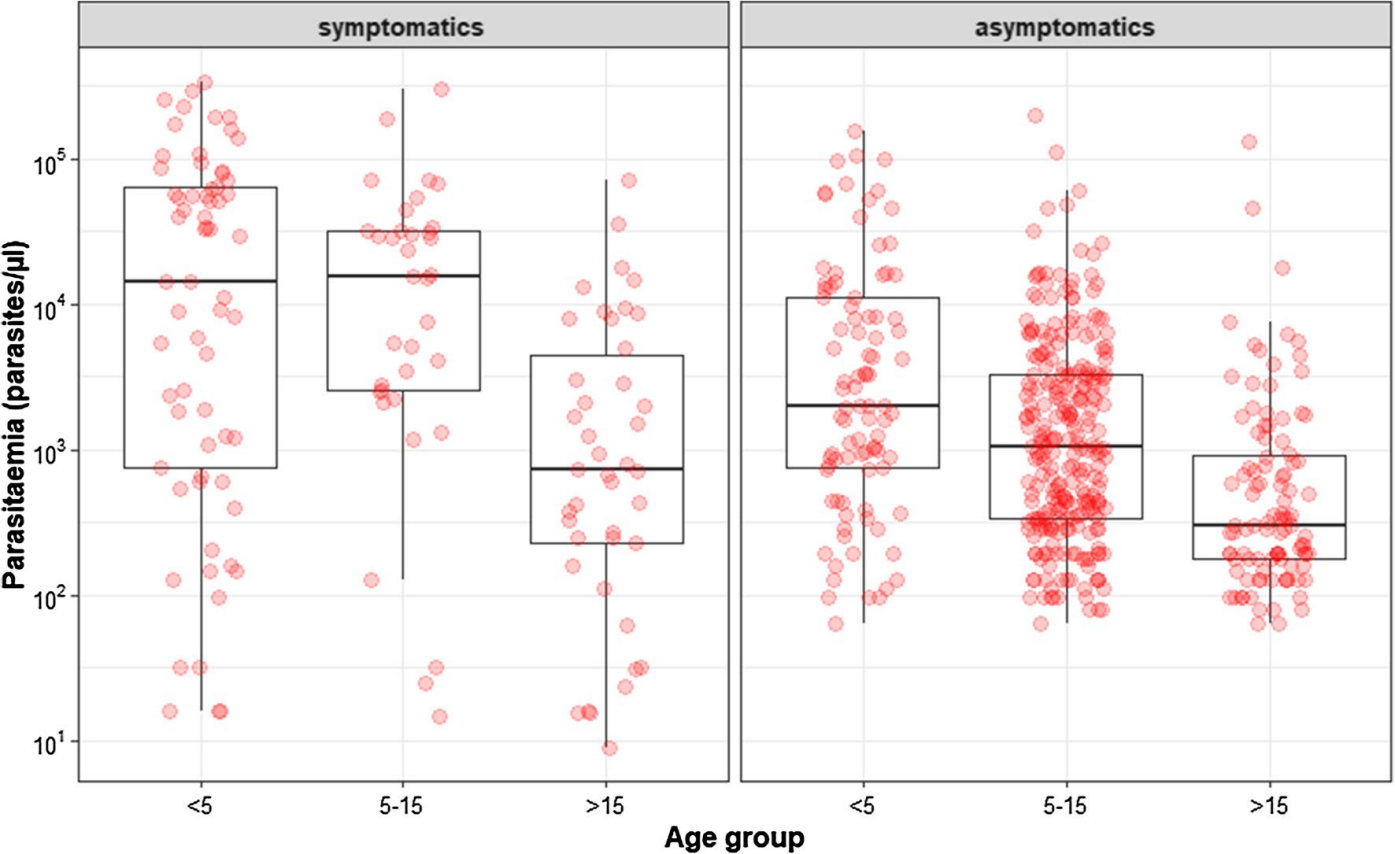
Boxplot of parasite densities for each age group in symptomatic and asymptomatic individuals

The median age of participants was 12 years (range 21 days to 85 years). The sex ratio (male/female) was 0.7 (558/809). Mean parasite densities were significantly higher in symptomatic patients from the health centers than in asymptomatic individuals recruited in the community, for both sexual (6199 ± 2626* vs* 38.75 ± 7.85, Mann–Whitney *U* = 1992; *P* < 0.0001) and asexual stages (34,164 ± 5332* vs* 5964 ± 867.8; Mann–Whitney *U* = 22,168; *P* < 0.0001). The mean asexual parasite densities significantly decreased with age in blood samples from both symptomatic and asymptomatic participants (Kruskal–Wallis test, *P* < 0.001; Fig. 2).

Of the 1367 samples, malaria diagnostic tools revealed 752 (55.01%) infections with *Plasmodium*. The infection rate was 41.8% (175/418) in samples from symptomatic patients and 60.8% (577/949) in samples from asymptomatic individuals, which is significantly different (*X*^2^ = 41.21;* P* < 0.0001). *Plasmodium falciparum* was the most prevalent *Plasmodium* species collected during both collection periods and represented 98.8 and 92.9% of human infections in 2015 and 2017, respectively. *Plasmodium malariae* and *P. ovale* each accounted for 0.6% of clinical infections. In comparison, in samples from asymptomatic persons a higher prevalence was recorded for *P. malariae* (22.9%) while *P. ovale* was rare (0.9%). Mixed infections were only found in asymptomatic carriers and were mostly represented by *P. falciparum*–*P. malariae* (92/96, 95.8%). No *P. vivax* infection was detected in any blood sample.

Malaria prevalence decreased with age in both asymptomatic individuals and symptomatic patients, and individuals older than 15 years were less infected (*χ*^2^ = 48.39 and *χ*^2^ = 49.91 respectively; *P* < 0.001). Gametocyte carriage was significantly higher in asymptomatic individuals than in symptomatic patients (16.2 *vs* 10.7%, *χ*^2^ = 6.96, *P* = 0.008). Heterogeneity in malaria infection was observed between neighborhoods, with infection rates varying from 48.2 to 71.2% inSabongari and Ngaoubela, respectively (*P* = 0.011; Table 2). A total of 53% (503/949) of asymptomatic carriers reported using LLINs and the coverage of LLINs varied from 21.1% in Yoko to 88% in Meng (Table 2). LLIN use based on self-report only conferred slight protection: malaria infection among LLIN users was only 7% less than that among non-users (non-significant difference; RR = 0.89, 95% confidence interval [CI] 0.58–0.98); however, the difference varied with collection site, with up to 20% protection found in Malarba.

**Table 2:** Malaria prevalence, long‐lasting insecticidal net usage and infection rate of mosquitoes according to neighborhoods in 2017

Neighborhoods	Blood samples		LLIN usage		Mosquitoes	
	*N*	IR (%)	Use	Frequency (%)	*N*	IR (%)
Malarba	150	66.0	46	30.7	40	2.5
Meng	183	61.2	161	88.0	63	1.6
Ngaoubela	111	71.2	77	69.4	78	1.3
Sabongari	83	48.2	48	57.8	3	0
Tella	100	57.0	39	39.0	0	0
Tomi	107	65.4	45	42.1	24	4.2
Wourtabbal	66	53.0	56	84.8	0	0
Yoko	147	57.1	31	21.1	96	5.2
Total	947	60.8	503	53.0	304	2.9

*IR* *Plasmodium* infection rate,* LLIN* long‐lasting insecticidal nets, *N* number of samples collected

### Mosquito species composition

**Fig. 3:**
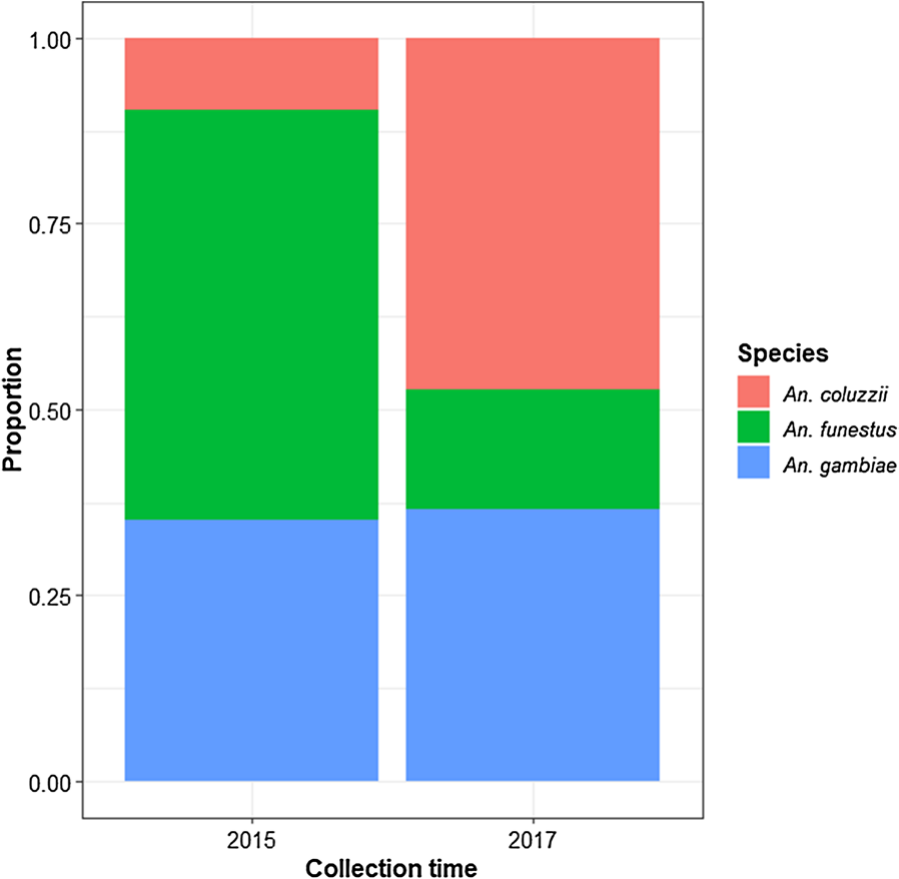
Distribution of *Anopheles* mosquitoes according to collection time

A total of 592 *Anopheles* were collected during both study periods. According to morphological criteria, the two species complexes identified were *An. gambiae* (*s.l.*) and *An. funestus* (*s.l.*). Molecular identification of specimens of *An. gambiae* sibling species showed that this fauna was composed of two species: *An. gambiae* (212; 35.8%) and *An. coluzzii* (172; 29.1%). *Anopheles funestus* (*s.s.*) was the only species identified within the *An. funestus* group and represented 35.1% (208/592) of anopheline mosquitoes. The distribution of the three species according to the two collection periods is shown in Fig. 3. No hybrid between *An. coluzzii* and *An. gambiae* mosquitoes was detected.

### Vector infection rates

**Fig. 4:**
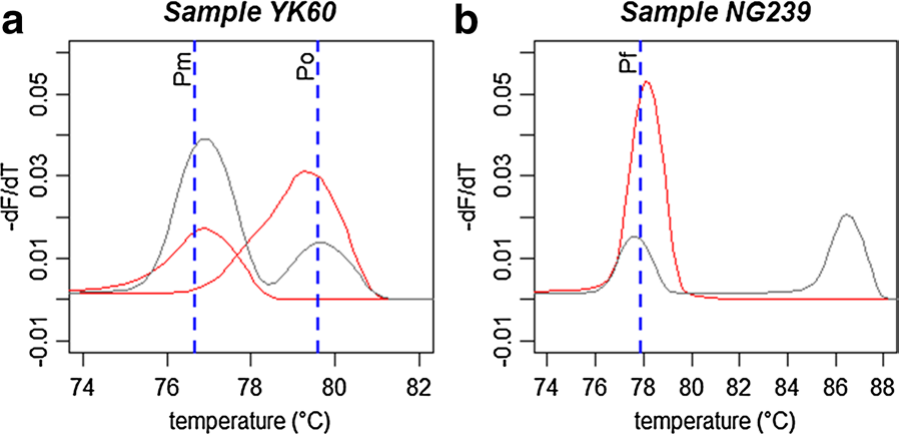
Melting curve peak of the YK60 and NG239 samples identified as being infected by a *Plasmodium* sp. The* x*-axis represents the melting temperature (Tm) and the* y*-axis represents the results of quantitative PCR on the Lightcycler real-time PCR system (*dF*/*dT* negative derivative of the fluorescence/derivative temperature). Each red and blue curve represents the Tm of each *Plasmodium falciparum*-positive control. The green curve represents the Tm of the sample

Head-thoraces of the 592 *Anopheles* were subjected to qPCR to detect infected mosquitoes. The results were obtained according to the melting temperature value given by an optical reading of the corresponding melting curve. In total, 26 (4.39%) *Anopheles* mosquitoes were infected by a *Plasmodium* sp., of which 24 could be clearly identified according to melting temperature. However, the remaining two samples (YK60 and NG239) gave a bimodal curve and were assigned as putative co-infection. YK60 was suspected to be *P. malariae*/*P. ovale* co-infection, with the main peak assigned to *P. malariae* and the minority peak to *P. ovale* (Fig. 4a). Further sequencing analysis identified the YK60 sample as a *P. malariae* monoinfection. For the NG239 sample (Fig. 4b), the melting temperature of the minority peak was assigned to *P. falciparum*, while the main peak did not match any *Plasmodium* sp. according to Mangold et al. [57]. The sequencing analysis revealed that the NG239 sample was a *P. falciparum* monoinfection.

The overall infection rate varied from 4.07 to 4.72% between mosquito species, and no significant difference was observed between species (*χ*^2^ = 0.046, *P* = 0.97; Table 3). The infection rate did not differ between the two collection periods (*χ*^2^ = 3.049, *P* = 0.08). Variation in mosquito infection rate was observed between neighborhoods in 2017, with Yoko (5/9) recording the highest number of infected mosquitoes (Table 3).

**Table 3:** Number of mosquitoes collected and of *Plasmodium*-positive mosquitoes according to quantitative PCR

*Anopheles* spp.	June 2015							July 2017						Total		
	Mosquitoes			*Plasmodium * sp.				Mosquitoes			*Plasmodium * sp.					
	*N*	*N*+	%	*Pf*	*Pm*	*Po*	*Pv*	*N*	*N* +	(%)	*Pf*	*Pm*	*Po*	*N*	*N*+	%
*An. gambiae*	101	7	6.93	4	3	0	0	111	3	(2.7)	2	0	1	212	10	4.72
*An. coluzzii*	28	2	7.14	1	0	0	1	144	5	(3.47)	2	1	2	172	7	4.07
*An. funestus* (*s.s.*)	159	8	5.03	6	1	1	0	49	1	(2.04)	1	0	0	208	9	4.33
Total	288	17	5.9	11	4	1	1	304	9	(2.96)	5	1	3	592	26	4.39

*N* Number of collected mosquitoes, *N+* number of *Plasmodium*-positive mosquitoes, *%* percentage of infected mosquitoes, *Pf Plasmodium falciparum*, *Pm* *Plasmodium malariae*, *Po* *Plasmodium ovale*,* Pv Plasmodium vivax*

All *Plasmodium*-positive specimens were monoinfected, either with *P. falciparum* (61.5%), *P. malariae* (19.2%) or *P. ovale* (15.4%). A single sample was found by qPCR to be positive for *An. coluzzii* with *P. vivax*.

**Fig. 5:**
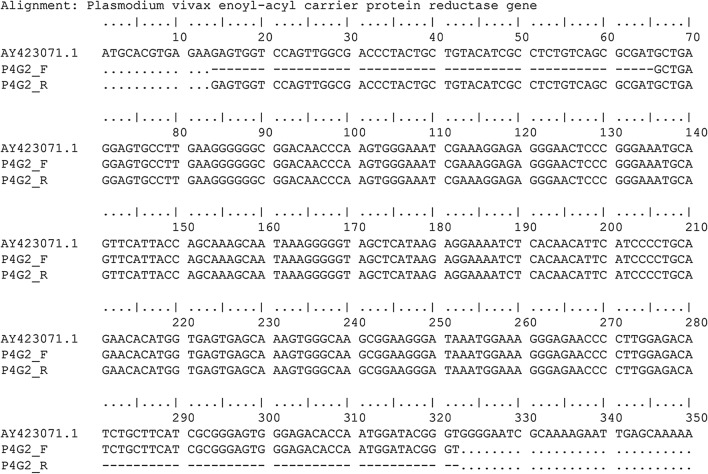
DNA sequence alignment of the *P. vivax* sample isolated from an infected *Anopheles coluzzii* sampled in 2015, referred to as sample P4G2, with the reference DNA sequence Pv_ECPR (accession number AY423071.1). P4G2_F and P4G2_R represent the forward and reverse sequences, respectively. Numbering corresponds to that of the Pv_ECPR sequence. No mismatchs were found

All *Plasmodium*-positive mosquitoes were processed for sequencing, and the sequence alignment of the *P. vivax* infection is presented in Fig. 5.

## Discussion

This study was performed to assess epidemiological and entomological parameters of malaria in Tibati, a locality situated in the Adamawa region of Cameroon. At the present time, little data exist on malaria in this area.

The incidence of malaria is known to vary according to epidemiological settings, age and clinical status of the study population and season, as well as with treatment guidelines and control interventions. Malaria prevalence in our study area varied from 42% in symptomatic patients to 61% in asymptomatic individuals in the community. This prevalence value is 15-fold higher than that reported by Songue et al. [25] in Meidougou, a village located within the same Adamawa region, where the authors reported a 3.6% infection rate based on microscopy studies of symptomless children aged < 10 years [25]. Other studies in Bolifamba, an area with a similar epidemiological setting, have identified an infection rate of > 50% by microscopy [58]. Malaria prevalence in studies in which malaria infections were diagnosed using PCR methodology also show large variations, with Fru-Cho et al. [28] reporting a malaria prevalence of 32% among asymptomatic carriers in the village of Bolifamba, and Russo et al. [29] reporting a 14% prevalence among febrile outpatients in Dschang, an altitudinal city in the West region. Although microscopy remains the “gold standard” tool for diagnosing malaria in the field, this approach has certain limitations, including inaccurate quality assurance and misdiagnosis of low parasitemia as the detection threshold by microscopy is about 50 parasites/µl. PCR-based methods are more sensitive and specific than the microscopic examination of thick blood smears, particularly in cases of low-density or mixed parasite infections that are frequent in asymptomatic carriers [59, 60]. Accordingly, 9% of the negative blood smears by microscopic examination were found to be positive upon molecular analysis in our study.

Almost half (42%) of the patients who went to the health care centers for consultation/treatment were diagnosed with malaria infection. Malaria remains the primary cause of fever in febrile patients, but other infections, such as Dengue, Chikungunya or Zika, likely also circulate in the area. Indeed, clinical symptoms of malaria are non-specific, and inter-epidemic arboviral infections are regularly reported in central Africa [61–64]. Accurate diagnosis of *Plasmodium* is therefore crucial for providing appropriate treatment regimens to patients. Determining the burden of malaria is also important for applying effective malaria control strategies in endemic settings and for determining their efficacy. In Cameroon, a nation-wide mass distribution campaign of LLINs took place in 2011, 2015 and 2019, and the NMCP reports a LLIN coverage of > 80% [65]. In our study, 53% (503/949) of asymptomatic individuals reported owning a bed net, but of these, 57.5% (289/503) were infected with malaria. It has been reported earlier that bed net coverage is not associated with asymptomatic *P. falciparum* prevalence [66]. It must also be considered that bed net coverage may be less than that acknowledged by the volunteers in our study. Barriers to mosquito net use have been described in previous studies, and a larger sensitization of households would be necessary to prevent the continuous spread of malaria [67, 68].

Asymptomatic infections represent the major reservoir of parasites [69]. In our study, we identified asymptomatic infection in 60.8% of blood samples collected from individuals at the community level, which indicates that malaria transmission is high in the area. Asymptomatic infections are associated with parasite exposure and decrease with age as prolonged exposure to malaria leads to the development of partial immunity to the disease [69, 70]. We noted that asymptomatic carriage was higher in the age group 5–15 years, which is in line with the development of acquired immunity in the older children. The lower prevalence of asymptomatic infections among individuals aged > 15 years likely represents the increased ability of older individuals to control parasite growth due to stronger anti-parasite immunity. However, some infections in this age group were certainly missed because asymptomatic infections are often low-density infections and undetectable through microscopy examination and even PCR testing [66, 69, 71]. Accordingly, parasite densities decreased with age in asymptomatic individuals. In symptomatic patients, lower parasite loads were found in the older age group (> 15 years), but densities were similar in children aged < 5 years and those aged 5–15 years, indicating that the parasite threshold to develop malaria symptoms is lower in older individuals, as previously observed [72]. Parasite burden contributes to the pathogenesis of the disease, and pyrogenic thresholds of parasitemia are often used to correlate fever with malaria, with thresholds varying with transmission intensity and age [73, 74]. Spatial heterogeneity in malaria infections was observed in our study, with Yoko presenting the highest malaria prevalence. This finding suggests hotspots of transmission in our study area. Multiple factors can explain this heterogeneity, such as proximity to breeding sites, urbanization or housing characteristics [75–77]. It is tempting to state that such high transmission clusters should be the focus of targeted control, but past interventions targeting hotspots of transmission failed to influence malaria transmission dynamics [78].

In this study, we identified three *Plasmodium* species in human blood samples, among which *P. falciparum* was the most predominant vector species, accounting for over 95% of malaria infections*.* These data are consistent with previous reports in other epidemiological settings, indicating that *P. falciparum* predominates across Cameroon [29, 79, 80]. *Plasmodium malariae* and *P. ovale* were found as mono- and co-infections with *P. falciparum*, but at low prevalence and these species were rarely found in symptomatic individuals. In the present study, no *P. vivax* infection was detected either in symptomatic or asymptomatic samples. Circulation of *P. vivax* in Cameroon has been recorded since 2014 in different epidemiological settings, even though previous reports documented *P. vivax* infections only in travellers returning from Cameroon [81, 82]. In previous reports, *P. vivax* infections in Cameroon were mostly identified at low frequencies, representing < 15% (13/269) of malaria infections in Bolifamba, southwest region [28], 6% (27/484) in Dschang, west Cameroon [29] and 4% (8/201) in five forested south parts of the country [27]. The latest study reported 23% (10/43) of *P. vivax* infections among symptomatic patients seeking medical attention at the New Bell district hospital in Douala, Littoral region [30]. Our failure to detect *P. vivax* in the present study suggests that the parasite circulates at very low frequency; as such, larger samples would be necessary to confirm this species in the area. The prevalence of *P. vivax* in Africa differs between epidemiological settings, and it has been suggested that *P. vivax* is more prevalent in areas with lower malaria burden [83].

We performed entomological surveys to identify mosquito vectors responsible for malaria transmission in Tibati. To date, six main vector species, namely *An. gambiae*, *An. coluzzii*, *An. funestus*, *An. arabiensis*,* An. nili *and* An. moucheti moucheti*, have a recognized role in *Plasmodium* transmission in Cameroon [2, 6, 84]. In the present study, three of these species were identified: *An*. *funestus*,* An. gambiae* and *An. coluzzii*. These data are consistent with previous reports that indicated a large distribution of these three main vectors across the country [6, 15, 85–89]. *Anopheles funestus* was more prevalent in 2015 than in 2017 (55.2* vs* 16%, respectively), and the change in the density of this species is likely due to modifications of environmental cues, such as the presence of permanent breeding sites (e.g. lakes and rivers with emergent vegetation) that are favorable for the proliferation of this mosquito species[49, 90]. This observed decrease in the density of *An. funestus* mosquitoes benefited *An. coluzzii*, which represented 9.7% of collected anophelines in 2015 and 47.4% in 2017. The presence of *An. gambiae* was stable over the two collection periods. The study area is irrigated by the Meng river and is characterized by the presence of agricultural and fish farming sites that create temporary sites which are favorable for the development of *An. gambiae* (*s.l.*), including *An. gambiae* and *An. coluzzii* [91, 92]. *Anopheles coluzzii* has a known high capacity of adaptation in urban environments [93–95], and modifications to the habitat in Tibati (e.g. deforestation, house construction, home improvement) that occurred during the 2 years between the studies have probably contributed to the creation of breeding sites more suitable for *An. coluzzii.* Nevertheless, the use of the IRS method, which is a widely used method for sampling endophilic mosquitoes, could have favored the collection of endophilic resting* Anopheles* over other mosquito species.

We found that *Plasmodium* infection rates did not differ between *An. gambiae*, *An. coluzzii* and *An. funestus*, and thus these three species are considered to transmit malaria at equal efficiency. Sporozoite infection rates fell within the range reported in a previous study conducted in the Adamawa region [87] and in other studies conducted in different parts of the country [2, 84]. A slight decrease in the overall infection rate was observed between 2015 and 2017 (5.9* vs* 4.39%; *X*^2^ = 2.791, *P* = 0.0948); this reduction, although not significant, could be due to the use of LLINs following the massive country-wide distribution campaign in 2015 [96]. The percentage of infected mosquitoes sampled in 2017 varied between neighborhoods, with the highest infection rate recorded in Yoko (5.2%), which could be explained by a high malaria prevalence and the lower LLIN coverage (21.1%) in this neighborhood.

All three mosquito species were mono-infected with *P. falciparum*, *P. ovale*,* P. malariae* and *P. vivax.* A single *An. coluzzii* mosquito was carrying *P. vivax* and the fact that only one mosquito was infected by *P. vivax* confirms the low circulation of this species in our study area. Further longitudinal studies or more in-depth sampling would increase the probability to detect *P. vivax* infections in both human and mosquito populations, but it remains that this one mosquito represents the first report of *Anopheles* infection by *P. vivax* in Cameroon. Transmission of *P. vivax* by anopheline mosquitoes has already been reported in sub-Saharan Africa, but never by *An. coluzzii*. Mosquito infections with *P. vivax* have been reported for *An. arabiensis* in Mauritania [97], Madagascar [98, 99] and Ethiopia [100], for *An. funestus* in Madagascar [98, 101], Kenya [102] and for *An. gambiae* in Kenya [102]. Also, a study conducted in Angola [41] and Equatorial Guinea identified *P. vivax* infections in mosquitoes, but identification of the vector species was not recorded [41]. Interestingly, in this latter study, the authors reported a significantly higher prevalence of *P. vivax* infection in mosquitoes (10.99%) as compared to human population (4.55%) [41]. The *P. vivax-*infected mosquito we identified was an *An. coluzzii* specimen, a species that has shown greater susceptibility to malaria infection in a study from Cameroon [55]. *Plasmodium vivax* can then be considered to circulate in the study area and may also be underestimated, as a proportion of *P. vivax* infections in humans are “hidden” since hypnozoites lie dormant in the liver for several months (or years) where they are undetected.

A limitation to this study is that mosquito samples were obtained at a single collection time and, therefore, the results only provide a one-shot picture of malaria transmission in the study site. Longitudinal surveys would be necessary to follow the dynamics of malaria transmission and, particularly, will be crucial to perform parasitological and entomological surveys before and after the implementation of control interventions, such as LLIN distribution. A second limitation is that sample sizes were small, which is possibly the reason we did not detect *P. vivax* infection in the human population. Regular monitoring of malaria infections will be necessary to assess the true circulation of *P. vivax* in the area; we cannot exclude the possibility that the *P. vivax*-positive mosquito got infected while feeding on a non-resident as Tibati is a cross-border city with a high circulation of people.

## Conclusion

We have provided a picture of the epidemiological and entomological malaria situation in Tibati, a small town in the Adamawa region of Cameroon. Malaria prevalence varied from 42% in symptomatic patients to 61% in asymptomatic individuals, and this finding highlights the high malaria endemicity in the area. Three major vectors, namely *An. funestus*, *An. gambiae* and *An. coluzzii*, were responsible for the transmission of the disease, with all three species contributing equally to *Plasmodium* transmission. Parasitological indices suggest low bednet usage and that the implementation of control interventions in the area is needed to reduce the malaria burden. We identified hotspots of disease transmission, and these sites should be the target of malaria control efforts. In addition, the presence of *P. vivax* in an *An. coluzzii* mosquito prompts for regular monitoring as the spread of this species could introduce further complexity into malaria epidemiology and control measures in this area.

## Data Availability

All data generated or analyzed during the current study are included in this published article.

## Abbreviations

CTAB: Cetyltrimethyl ammonium bromide
IRS: Insecticide residual spraying
LLINs: Long‐lasting insecticidal nets
MINSANTE: Ministère de la Santé
PCR‐RFLP: PCR‐restriction fragment length polymorphism
rRNA: Ribosomal ribonucleic acid
