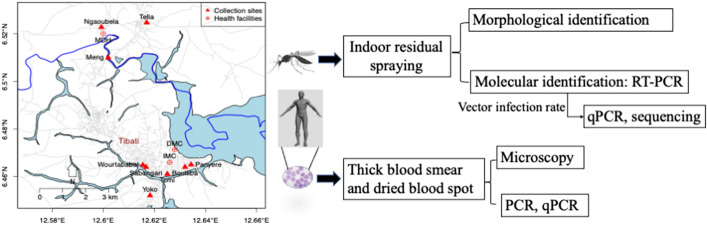# Correction to: Epidemiological and entomological studies of malaria transmission in Tibati, Adamawa region of Cameroon 6 years following the introduction of long-lasting insecticide nets

**DOI:** 10.1186/s13071-021-04912-1

**Published:** 2021-10-04

**Authors:** Lionel Brice Feufack-Donfack, Elangwe Milo Sarah-Matio, Luc Marcel Abate, Aline Gaelle Bouopda Tuedom, Albert Ngano Bayibéki, Christelle Mafo Ngou, Jean-Claude Toto, Maurice Marcel Sandeu, Carole Else Eboumbou Moukoko, Lawrence Ayong, Parfait Awono-Ambene, Isabelle Morlais, Sandrine Eveline Nsango

**Affiliations:** 1 Service de Paludisme du Centre Pasteur Cameroun, BP 1274, Yaounde, Cameroon; 2 CNRS UPR 9022, Inserm U 963, Université de Strasbourg, 2, allée Konrad Roentgen, 67084 Strasbourg Cedex, France; 3 UMR MIVEGEC, Institut de Recherche Pour Le Développement, IRD, CNRS, Université de Montpellier, 911 avenue Agropolis, 34394 Montpellier, France; 4 Université Catholique D’Afrique Centrale, Yaoundé-Campus Messa, BP 1110, Yaounde, Cameroon; 5 Laboratoire de Recherche Sur Le Paludisme, Organisation de Coordination Pour La Lutte Contre Les Endémies en Afrique Centrale, BP 288, Yaounde, Cameroon; 6 Department of Medical Entomology, Centre for Research in Infectious Diseases, Yaounde, 13591 Cameroon; 7 Department of Microbiology and Infectious Diseases, School of Veterinary Medicine and Sciences, University of Ngaoundere, PO Box 454, Ngaoundere, Cameroon; 8 Faculté de Médecine Et Des Sciences Pharmaceutiques de L’Université de Douala (FMSP–UD), BP 2701, Douala, Cameroon

## Correction to: Parasites Vectors 14:247 (2021) https://doi.org/10.1186/s13071-021-04745-y

Following publication of the original article [1], it came to our attention that the article had published with an incorrect file for the Graphical abstract; the article had published with a screenshot of its Abstract as the Graphical abstract.

The original article has since been updated and the correct Graphical abstract may be found in this correction for reference.